# Establishment of an iodine model for prevention of iodine-excess-induced thyroid dysfunction in pregnant women

**DOI:** 10.1515/biol-2021-0142

**Published:** 2021-12-31

**Authors:** Yuhan Zhou, Fen Chen, Lingyu Wang, Chunhui Tian, Shuo Zhang, Feifei Ding, Jie Deng

**Affiliations:** Department of Obstetrics, Xiangyang No. 1 People’s Hospital, Hubei University of Medicine, No. 15 Jiefang Road, Fancheng District, Xiangyang, 441000, People’s Republic of China; Department of Technology, Biotecan Medical Diagnostics Co., Ltd, Zhangjiang Center for Translational Medicine, Shanghai 201204, People’s Republic of China

**Keywords:** urinary iodine concentration, pregnant women, a logistic regression model, thyroid dysfunction

## Abstract

This study aims to explore the relationship between the iodine status and thyroid dysfunction (TD) in pregnant women and establish a model to guide them to prevent excessive iodine intake. A total of 515 pregnant women were enrolled in the study. Urinary iodine concentration (UIC), thyroid hormones, and thyroid autoantibodies were measured, and then a logistic regression model was established. The median UIC of pregnant women was 174 ± 120 μg/L. Multivariate logistic regression analysis indicated that multivitamin supplements containing iodine and frequent seafood consumption were risk factors for excessive iodine (UIC ≥500 μg/L). Besides, excessive iodine was a risk factor for TD. Iodine excess was associated with a high prevalence of TD in pregnant women, especially TPOAb-positive women (*P* < 0.05). A logistic regression model based on potential risk factors was established to predict the risk of excessive iodine intake among pregnant women and provide guidance to minimize the risk of excessive iodine intake, thus reducing the risk of TD.

## 1 Introduction

The synthesis of thyroid hormones requires iodine, which plays a key role in maintaining basic metabolism and body growth. Diet and drinking water are the main sources of iodine. Urinary iodine concentration (UIC) is an important biomarker of iodine metabolism [1]. The iodine demand of pregnant women is 50% higher than that of nonpregnant adults because active transport of iodine through the placenta to the fetus, the increase of thyroid hormone production, and the increase of glomerular filtration. All of these will lead to iodine loss, which increases the risk of iodine deficiency in pregnant women [2,3,4]. Significant changes in iodine metabolism during pregnancy have a vital impact on thyroid function [5]. Thus, thyroid disorders are more common in pregnant women [6,7]. Insufficient iodine intake may lead to maternal thyroid dysfunction (TD) and adversely affect fetal nerve development [8].

China is known as the iodine deficiency area due to the lack of environmental resources. Xiangyang City, the second-largest city in Hubei Province, located in central China, has been proved to be an iodine-deficient area. Since the 1990s, most countries, including China, have implemented universal salt iodization (USI), which has greatly improved iodine nutrition in the world [9,10]. In the past two decades, China has almost eliminated iodine deficiency disorders (IDDs) [11,12,13]. With increasing iodine levels in pregnant women in inland China, the risk of iodine excess has also attracted great attention. Though previous studies have well explained the relationship between iodine status in pregnant women and TD, there are only a few reports on how to control multiple risk factors scientifically to prevent iodine excess and TD. Clinical factors, such as iodized salt, iodine-containing supplements and dietary intake, are considered as risk factors for iodine excess. Although World Health Organization (WHO) recommends UIC to represent the iodine status in the body, the best method to assess iodine status remains controversial. Furthermore, the random urine iodine cannot fully tell us whether the amount of iodized salt or iodine-rich diet is appropriate and how to adjust the diet of pregnant women; therefore, it is meaningful to predict the high-risk group of pregnant women with more than adequate iodine or excessive iodine intake through multiple variables, especially for pregnant women who generally eat iodized salt in central China. They need to be cautious about taking iodine supplements or food to reduce the risk of TD-related iodine excess.

This study aimed to explore the relationship between iodine status and TD in Chinese pregnant women. A model was constructed to screen pregnant women with a high risk of iodine excess based on potential risk factors and help them supplement iodine properly to prevent TD-related iodine excess.

## 2 Materials and methods

### 2.1 Subjects

A total of 515 pregnant women were enrolled from the obstetrics department of the No. 1 People’s Hospital of Xiangyang City, Hubei Province, China, from October 2018 to January 2020. The inclusion criteria were as follows: (1) no history of a primary and metastatic tumor; (2) no history of any drug influencing thyroid function; (3) no history of thyroidectomy; and (4) complete laboratory examination or questionnaire. Clinical information including age, gestational week, prepregnant body mass index (BMI), dietary habits, and exogenous iodine supplementation during pregnancy were filled in. After three experts reviewed the clarity and understanding of the questionnaire, the trained nurses collected data through face-to-face interviews.


**Informed consent:** Informed consent has been obtained from all individuals included in this study.
**Ethical approval:** The research related to human use has been complied with all the relevant national regulations, institutional policies and in accordance with the tenets of the Helsinki Declaration, and has been approved by the Research Ethics Committee of the Hubei University of Medicine.

### 2.2 Iodine supplementation

Dietary habits, such as seafood (three groups: no seafood consumption; occasional: 1–2 meal containing seafood per week on average; frequent: at least three meals containing seafood per week), egg (three groups: no egg consumption; occasional: 1–2 eggs per week on average; frequent: at least three eggs per week), cow’s milk and yogurt (three groups: no consumption; occasional: 1–500 mL per week on average; frequent: at least 500 mL per week), were recorded according to the above grading rules. In 2011, the Ministry of Health issued the “iodine content of edible salt,” which stipulated that the average level of iodine content in edible salt can be 20, 25, and 30 mg/kg. One or two kinds of iodized salts can be supplied according to the iodine nutrition level of people in the province. Xiangyang Municipal governments used iodized salt with 25 mg/kg iodine standard. The iodized salt used for cooking by pregnant women is defined as the use of iodized salt, and those without iodized salt for cooking are defined as no iodized salt use. The doctor prescribed multivitamin tablets to pregnant women with vitamin deficiency. According to the doctor’s advice, pregnant women took one or two tablets daily (50 or 100 µg of iodine intake). Iodine-containing multivitamin supplements taken by pregnant women are referred to as iodine supplements. Pregnant women who do not take a multivitamin supplement or take non-iodine-containing multivitamin supplements are defined as iodine-free supplements.

### 2.3 Measurement of thyroid functional parameters

The blood samples of each pregnant woman were drawn into the serum separator tubes, and the serum was centrifuged. Serum-free thyroxine (FT4), free triiodothyronine (FT3), thyroid-stimulating hormone (TSH), and thyroid peroxidase antibody (TPOAb) were measured by an automatic biochemical analyzer (Abbott Laboratories, Chicago, USA). The normal values were determined by the 5th and 95th percentiles of serum measurement. Table S1 shows the reference ranges of thyroid hormones.TD was diagnosed according to the following definitions:

(1) Clinical hyperthyroidism (any one of the below criteria): decreased TSH, elevated FT4, and/or elevated FT3; patients who had a history of hyperthyroidism were receiving anti-thyroid agents.
(2) Clinical hypothyroidism: elevated TSH and decreased FT4; patients who had a history of hypothyroidism were receiving levothyroxine.
(3) Subclinical hyperthyroidism: decreased TSH and normal FT4.
(4) Subclinical hypothyroidism: elevated TSH and normal FT4.

### 2.4 UIC detection

A fresh spot urine sample (10 mL) from each pregnant woman was collected between 8:00 and 12:00 a.m. Urine samples were stored at 4°C and tested within 8 h after receiving the samples. They were detected in duplicate by inductively coupled plasma mass spectrometry (Agilent Technologies, Inc., Tokyo, Japan). Self-made quality control material was used in every run and the blank solution was routinely prepared and tested to monitor potential cross-contamination in urine samples. The authors of this project participated in the External Quality Assessment organized by the National Health Commission of the People’s Republic of China. The iodine status of pregnant women is divided into four grades according to the WHO criteria, including insufficient (mUIC <150 μg/L), adequate (mUIC, 150–249 μg/L), above requirements (mUIC, 250–499 μg/L), and excessive (mUIC ≥500 μg/L) intake.

### 2.5 Statistical analysis

Data were analyzed by using Python software (Version 3.6) (https://www.python.org). Categorical variables were presented as numbers with proportions (%). Normal distribution was tested using the Shapiro–Wilk test. Continuous variables were shown as median with the interquartile range (IQR). The Kruskal–Wallis *H* test and Chi-square test were used to calculate intra-group or inter-group statistical differences. Multivariate logistic regression analysis was used to identify the risk factors for iodine excess or TD. A logistic regression model was established to predict the probability of iodine excess. The predictive value was evaluated through the receiver operating characteristic curve. The model was verified by random sampling of more than 10×. *P*-value <0.05 was deemed as a statistically significant difference.

## 3 Results

### 3.1 General characteristics of pregnant women

The median UIC of pregnant women with TD was 199 ± 121 μg/L, higher than that of pregnant women without TD (*P* = 0.034). The FT4 level in the TD group was significantly higher than that in the euthyroid group (*P* = 0.032). The proportion of TPOAb positivity in pregnant women with TD was higher than that in pregnant women with normal thyroid function (*P* = 0.008). Compared with the euthyroid group, the number of pregnant women who took iodine supplements in the TD group was more according to the questionnaire (*P* = 0.032). Pregnant women with TD consumed more iodine-rich food, such as seafood and milk, every week compared to pregnant women without TD. About 87% of pregnant women took iodized salt in this cohort (Table 1).

**Table 1:** General characteristics of pregnant women

	TD (*n* = 147)	Euthyroid (*n* = 368)	*P* value
Age (years)^*^	28.0 (26.5, 31.5)	28 (26.0, 30.0)	0.322
Gestational week (weeks)^*^	15 (13, 16)	15 (13, 16)	0.307
Prepregnancy BMI (kg/m^2^)^*^	22.2 (20.5, 24.6)	22.2 (20.2, 24.2)	0.834
UIC (μg/L)^*^	199 (139, 264)	171 (109, 257)	0.034
FT4 (pmol/L)^*^	12.6 (11.3, 13.6)	12.2 (10.9, 13.3)	0.032
FT3 (pmol/L)^*^	4.6 (4.2, 5.0)	4.5 (4.1, 4.9)	0.419
TSH (nIU/L)^*^	3.8 (0.3, 5.1)	1.8 (1.1, 2.5)	0.907
TPOAb positivity	23 (15.7)	29 (7.9)	0.008
Iodized salt			
Yes	133 (90.5)	318 (86.4)	0.255
No	14 (9.5)	50 (13.6)	
Multivitamin with iodine			
Yes	54 (36.7)	100 (27.2)	0.032
No	93 (63.3)	268 (72.8)	
Seafood (fish, crab, shrimp)			
Frequent	40 (27.2)	61 (16.6)	0.011
Occasional	89 (60.5)	270 (73.4)	
None	18 (12.2)	37 (10.1)	
Egg			
Frequent	80 (54.4)	158 (42.9)	0.056
Occasional	60 (40.8)	192 (52.2)	
None	7 (4.8)	18 (4.9)	
Cow’s milk			
Frequent	110 (74.8)	173 (47.0)	<0.0001
Occasional	24 (16.3)	169 (45.9)	
None	13 (8.8)	26 (7.1)	
Yogurt			
Frequent	42 (28.6)	78 (21.2)	0.057
Occasional	64 (43.5)	202 (54.9)	
None	41 (27.9)	88 (23.9)	

Categorical variables are presented as numbers with proportions (%) and are presented as median (IQR) (^*^).

Statistically significant difference set at *P* < 0.05, Kruskal–Wallis *H* test.

### 3.2 Maternal iodine status and TD risk

Pregnant women whose UIC was above 500 μg/L had the lowest FT4 level and highest TSH. All functional thyroid parameters were in the normal reference ranges (Table S2). Iodine excess (UIC ≥500 μg/L) acted as a risk factor for TD (OR, 1.02; 95% confidence interval [CI], 1.00–1.04, *P* = 0.019) while iodine deficiency was not a risk factor for TD after additional adjustment for other variables (Table 2).

**Table 2:** Association between UIC and TD risk

UIC (μg/L)	OR (95% CI)	*P*-value
0–149	1.00 (0.98–1.01)	0.041*
150–249	Ref	
250–499	1.00 (0.99–1.01)	0.018*
≥500	1.02 (1.00–1.04)	0.019*

Data presented as odds ratio (OR) (95% confidence interval [CI]).

*Statistically significant difference set at *P* < 0.05.

### 3.3 Risk factors for iodine excess of pregnant women

About 87.6% of pregnant women in this cohort consumed iodized salt, and a significant correlation between iodized salt intake and higher UIC was observed (*P* < 0.0001). The iodine-containing multivitamin supplement was significantly associated with higher UIC (*P* < 0.0001). Frequent seafood and milk intake contributed to higher UIC (*P* = 0.028 and *P* = 0.001, respectively) (Table S3). When we recalculated the OR value in the multivariable logistic regression model, we found that iodine supplement and seafood intake were with OR > 1 and *P* < 0.05 (Table S4). After additional adjustment for age, gestational week, and prepregnancy BMI, iodine-containing multivitamin supplement and frequent seafood intake were proved to be risk factors for iodine excess (Table 3).

**Table 3:** Multivariable logistic regression analysis of risk factors for excessive iodine intake after additional adjustment for age, gestational week, and BMI

Variables	β	SE	Wald *χ* ^2^	OR (95.0% CI)	*P*-value
Iodized salt	0.33	0.31	1.84	1.38 (0.27–1.77)	0.058
Iodine-containing supplements	0.41	0.36	1.78	1.51 (1.18–2.10)	0.011
Sea food	0.06	0.06	2.05	1.09 (1.02–1.56)	0.047
Egg	−0.02	0.02	−2.55	0.98 (0.90–1.01)	0.967
Milk	0.03	0.04	2.31	1.03 (0.99–1.12)	0.121
Yogurt	−0.13	0.16	−2.29	0.88 (0.62–0.99)	0.481

OR, odds ratio; CI, confidence interval.

### 3.4 Prevalence of TD according to UIC

The total prevalence of TD in the iodine excess group was much higher (52.94%) than that in inadequate and more than adequate iodine groups (26.95 and 25.19%, respectively). The proportion of TD in the UIC deficiency group was relatively higher than that in the adequate and more than adequate groups though there was no statistical difference. In TPOAb-positive pregnant women, the incidence of TD was closely related to UIC. The proportion of TD in the iodine excess group was the highest among TPOAb-positive pregnant women. However, this association was not seen in TPOAb-negative groups. Subclinical hyperthyroidism was the most common disorder of TDs in the UIC deficiency group, while subclinical hypothyroidism was most common in more than adequate and excessive iodine groups (Table 4).

**Table 4:** Prevalence of TD according to UIC

	TD *n* (%)						
	Deficiency (1)	Adequate (2)	More than adequate (3)	Excess (4)	*P*-value	*P*-value	*P*-value
	*n* = 200	*n* = 167	*n* = 131	*n* = 17		(2) vs (4)	(3) vs (4)
Total							
Euthyroid	140 (70.00)	122 (73.05)	98 (74.81)	8 (47.06)	0.107	0.025	0.017
TD	60 (30.00)	45 (26.95)	33 (25.19)	9 (52.94)			
TPOAb (+)							
Euthyroid	14 (7.00)	9 (5.39)	6 (4.58)	0	0.040	0.089	0.159
TD	4 (2.00)	8 (4.79)	8 (6.11)	3 (17.65)			
TPOAb (−)							
Euthyroid	25 (12.50)	16 (9.58)	15 (11.45)	2 (11.76)	0.150	0.281	0.402
TD	18 (9.00)	29 (17.37)	21 (16.03)	1 (5.88)			
Subtypes							
Hyperthyroidism	4 (2.00)	3 (1.80)	0	0	—	—	—
Hypothyroidism	2 (1.00)	3 (1.80)	1 (0.76)	2 (11.76)			
Subclinical hyperthyroidism	52 (26.00)	19 (11.38)	0	0			
Subclinical hypothyroidism	2 (1.00)	20 (11.98)	32 (24.43)	7 (41.18)			

Categorical variables are presented as numbers with proportions (%).

Statistically significant difference set at *P* < 0.05. Chi-square test for the difference among groups.

### 3.5 A prediction model of iodine overconsumption

**Figure 1:**
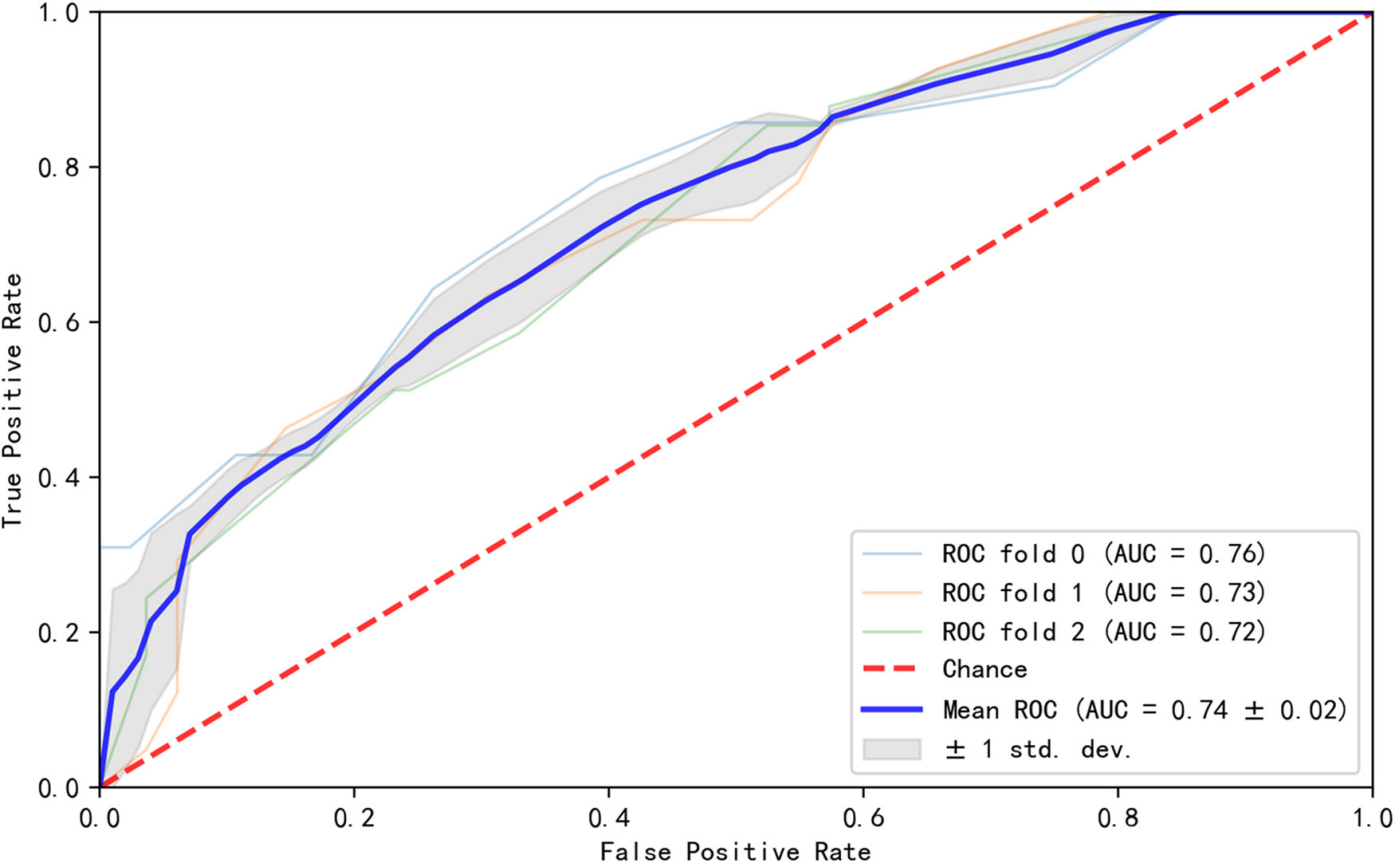
A prediction model of iodine excess among pregnant women. The AUC of the model was 0.74.

A logistic regression model based on iodized salt, multivitamin supplement with iodine, and iodine-rich diet intake for predicting iodine excess was built (AUC = 0.74) (Figure 1). The feature importance of the variables in the model showed that iodine-containing multivitamin was the most important factor for iodine excess, followed by iodized salt, seafood, and milk intake (Figure S1). The model was verified by sensitivity analysis (Figures S2 and S3). Based on the model, adjustable measures such as adjusting edible salt or multivitamin supplement to non-iodized ones or reducing iodine-rich food consumption can be recommended to pregnant women to prevent excessive iodine intake (Tables 4 and Table 5).

**Table 5:** Clinical suggestions through the model

Score	Risk	Adjustment suggestions
0.56–1.0	High	Not taking iodine-containing supplements and reducing iodine-rich food intake according to the model
0.26–0.55	Medium	Replacing iodized salt to non-iodized salt or reducing iodine-rich food intake according to the model
0.0–0.25	Low	Monitor UIC

## 4 Discussion

Since the implementation of USI in China in 1996, iodine nutrition has been greatly improved [14,15]. However, some regions in China still indicate the need to strengthen iodine supplementation to reach the WHO’s benchmark of 150 μg/L [16,17]. This study showed that the median UIC of pregnant women was 174 ± 120 μg/L in an iodine-deficient area, which was similar to the value previously reported in China [18,19]. About 87% of pregnant women in this cohort took iodized salt, which directly affected the increase of UIC of pregnant women in this inland area. Besides, the intake of iodine-rich food and multivitamin (iodine-containing) supplements was also closely associated with elevated UIC in pregnant women. Iodine excess caused by iodized salt in some areas may lead to TD in mothers and infants [20].

TD is the most common endocrine disorder during pregnancy [7]. We observed TD in 147 (28.54%) pregnant women. The proportion of pregnant women with iodine excess in the TD group was 4.76%, which was higher than 2.72% in the normal thyroid function group, indicating that iodine excess in pregnant women was associated with abnormal thyroid function. Our results showed that more TPOAb positivity was found in the TD group. Among pregnant women with TD, the number of people who like to eat seafood and milk was higher. We observed that long-term consumption of iodine-rich food was not related to elevated FT4, but daily consumption of iodized salt was negatively related to a decrease in FT4 levels. About 52.94% of the patients in the iodine excess group harbored TD, which was higher than that in the iodine adequate and more than adequate groups. TSH was found to have a positive correlation with UIC. Consistent with these, subclinical hypothyroidism was the most common disorder of TDs in the UIC excess group, while subclinical hyperthyroidism was most common in the UIC deficiency group. Especially in the TPOAb-positive pregnant women, the occurrence of TD was closely related to UIC. Generally, when the UIC level was higher than or even exceeded the requirement, TD was more likely to occur. In the iodine excess group, the proportion of pregnant women with TD was the highest. However, when pregnant women were negative for TPOAb, there was no linear correlation between TD and UIC. To sum up, these results showed that the intake of iodized salt and long-term consumption of iodine-rich food was helpful to improve the UIC of pregnant women and avoided the occurrence of IDDs, but at the same time, high UIC caused by exogenous iodine supplementation would directly affect the thyroid function of pregnant women. Therefore, in central China, where iodized salt is widely used, iodine supplementation during pregnancy should be done cautiously because excessive iodine will not only adversely affect the thyroid function of mothers but also affect the growth and development of newborns [21,22]. In response to the call of the Ministry of Health of the People’s Republic of China, who stipulates that the average level of iodine content in edible salt can be 20, 25, and 30 mg/kg. Xiangyang municipal government chose 25 mg/kg as the standard. Whether this dose is appropriate for each individual is still unknown. We calculated the OR value in the multivariable logistic regression model and found that two variables, multivitamin supplement with iodine and frequent seafood intake, with OR > 1 and *P* < 0.05, were regarded as risk factors for excessive iodine (UIC ≥500 μg/L).

The spot urine iodine test cannot fully reflect the iodine status in the body, nor can it suggest that it is appropriate to eat iodized salt or iodine-rich food. Therefore, it is meaningful to predict the high-risk population of iodine excess through multiple variables. Especially, pregnant women in central China generally eat iodized salt, so they need to pay attention to the amount of iodine-containing supplements or food to prevent excess. We suggest that the combination of multiple risk factors is more effective in predicting risk than a single biomarker such as random spot urinary iodine. To better guide the supplementation of iodine and avoid excessive iodine, we established a monitoring model. Taking the amount of iodine-containing supplements, the intake of iodized salt, and eating habits as variables, the risk level was deduced from the output value of the model. Pregnant women should replace iodine-containing supplements or salt with non-iodized-containing ones or reduce the frequency of iodine-rich food intake to prevent iodine excess, and the corresponding adjusted dose and frequency according to the risk range can be the output. For a pregnant woman whose UIC was above adequate, the model can evaluate the possible causes of high iodine status together with the UIC value and give adjustment measures. For a pregnant woman whose UIC was still in the normal range, the model can predict whether the pregnant woman is at risk of iodine excess based on her current iodine intake. Especially for TPOAb-positive pregnant women, this model can help them avoid iodine excess and minimize the occurrence of TD. These results show that adequate iodine intake and avoiding excessive iodine intake are of great significance for clinical monitoring of thyroid function in pregnant women. However, there are some limitations to this study. First, the sample from a certain region was relatively small. Second, when samples were collected at one time point, one test cannot represent the iodine status of the whole pregnancy, which would affect the statistical analysis. In a future study, a larger sample size is needed to verify these results.

## 5 Conclusion

In central China, where iodized salt is widely used, iodine-containing multivitamin supplements and seafood intake were the main risk factors for iodine excess. Iodine excess was associated with a high prevalence of TD in pregnant women, especially TPOAb-positive women. A model was established to help clinicians provide corresponding suggestions for rational iodine supplementation for pregnant women, especially TPOAb-positive women.
